# Challenging aged care stigma through communication: discursive responses to stigmatising discourses about aged care work and implications for workers’ mental health

**DOI:** 10.1007/s10433-025-00844-2

**Published:** 2025-04-03

**Authors:** Asmita V. Manchha, Ken Tann, Kïrsten A. Way, Michael Thai

**Affiliations:** 1 Queensland Aphasia Research Centre, School of Health and Rehabilitation Sciences, The University of Queensland, Brisbane, Herston, QLD 4029 Australia; 2 Surgical Treatment and Rehabilitation Service (STARS) Education and Research Alliance, The University of Queensland and Metro North Health, Brisbane, QLD Australia; 3 School of Health and Rehabilitation Sciences, The University of Queensland, Brisbane, Herston, QLD 4029 Australia; 4 School of Psychology, The University of Queensland, Brisbane, Herston, QLD 4029 Australia; 5 The University of Queensland Business School, Brisbane, QLD Australia

**Keywords:** Aged care work, Occupational stigma, Dirty work, Discursive responses, Mental health

## Abstract

Occupational stigma can negatively impact aged care workers’ (ACWs) mental health. This mixed-methods study investigates whether ACWs who challenge stigmatising discourses, through communication, experience reduced psychological costs of aged care stigma. We screened 184 ACWs’ discursive responses and tested for differences in ACWs’ mental health between those who challenge stigma and those who do not. A discourse approach was further employed to examine recurring language patterns in ‘*challenge*’ discursive responses. ACWs (*n* = 95) who discursively challenged stigmatising discourses reported lower internalised occupational stigma and psychological distress than those who did not challenge stigma (*n* = 89). These workers chose to infuse positive value into negative evaluations about ACWs and aged care work. Overall, findings suggest that ACWs may spontaneously challenge occupational stigma, through their discursive responses, which may proactively protect their mental health. We offer practical implications for challenging stigma, including developing guidelines, training, and language-based interventions.

**Supplementary Information:**

The online version contains supplementary material available at 10.1007/s10433-025-00844-2.

## Introduction

Studies have revealed that people employed in stigmatised occupations experience greater emotional exhaustion (Guerreo et al. 2021), depression (Benoit et al. 2015) and anxiety (Schubert et al. 2021) which in turn can drive greater turnover intentions (Labrague and de Los Santos 2021). In addition to individual harms, this may have substantial social and economic consequences, contributing to higher recruitment and training costs, placing additional strain on both public and private healthcare systems (Duffield et al. 2014). Addressing stigma may help mitigate poor mental health outcomes, which can improve staff retention, and ultimately foster broader financial and social benefits.

Although ample studies have examined the ways in which workers psychologically cope with stigmas surrounding their work (see Soral et al. 2021), findings have predominantly emphasised the difficulty in alleviating the negative impact of stigma on workers’ psychological wellbeing. Little work has explored whether there are differences between workers who spontaneously choose to challenge or not challenge occupational stigma when they are presented with a stigmatising discourse, in terms of psychological wellbeing. In the present study, we investigate aged care workers’ (ACWs) spontaneous discursive responses to stigmatising discourses surrounding aged care work (i.e. providing direct care to older adults) with discursive responses referring to the process of construing evaluations through interpersonal communication (Meisenbach 2010). Recent evidence suggests that ACWs are highly susceptible to psychological costs through performing tasks that society perceives as low status and “dirty work” (Manchha et al. 2022a). We draw on the occupational stigma literature (Ashforth and Kreiner 1999; Ashforth et al. 2007) and Stigma Theory (Yang et al. 2007) to investigate (a) whether ACWs discursively challenge or do not challenge stigmatising discourses surrounding their occupation and (b) the relationship between ACWs’ discursive responses to stigma (i.e. whether or not they challenge the stigma) and mental health outcomes (specifically, internalised occupational stigma and psychological distress). Internalised occupational stigma refers to the phenomenon whereby people diminish their own worth based on how others devalue their work (Major et al. 2018). Psychological distress refers to stress, anxiety, and depressive symptoms (Kessler et al. 2002).

### Occupational stigma and aged care work

We refer to occupational stigma as negative social evaluations that discredit people who work in occupations perceived to be tainted or flawed (Ashforth and Kreiner 1999). The physical nature of tasks (i.e. involving bodily functions, incontinence) and association with clients and colleagues who may belong to perceived low status groups, have construed a stigmatised image of aged care work (Ostaszkiewicz et al. 2016). Recent studies suggest this occupational stigma continues to reduce the social esteem of those who perform aged care work (Manchha et al. 2021; 2022a; 2022b). Scales et al. (2017) have also found that ACWs feel disempowered when they perceive that they work in a stigmatised occupation. While we acknowledge that aspects of aged care work such as work demands are associated with poorer mental health in ACWs (Gibson et al. 2022), some of these conditions can be difficult to change in the short-term. Thus, we postulate that communication strategies may provide more proximal approach to manage occupational stigma that is within the control of individual workers.

### Spontaneous strategies for challenging occupational stigma

Although working in a perceived stigmatised occupation is associated with negative consequences, researchers have argued that workers who are able to challenge stigma associated with their occupation report more positive psychological outcomes (Zhang et al. 2021). Workers may address internalised stigma using strategies targeted at a group and/or individual level. Collective strategies may involve group-based approaches which create supportive peer networks and foster strong collective identity. For example, Walsh et al. (2019) found that employees experienced improved organisational identification in the wake of their failed tech company when they re-affirmed their relevance and value in their current circumstances. Just and Muhr (2020) found pole dance instructors leveraged occupational pride to reduce tensions between feeling empowerment and occupational taint, when teaching students, to build collective feminine sexuality. Studies have also examined how workers may challenge stigma at an individual level (see Soral et al. 2021). These studies have been qualitative in nature and focus on asking workers directly about *how* they counter or overcome the negative effects of stigma. Stigmatised workers have been found to employ any combination of communication strategies (see Meisenbach 2010) including widely shared ideological techniques (such as reframing, recalibrating, refocusing), social buffers (e.g. comparing self to other groups who are worse off than them) and/or defensive tactics (e.g. avoiding/denying) in order to mitigate the stigma surrounding their occupation (Kreiner et al. 2021).

However, we argue that, in the absence of being explicitly asked to describe strategies they use to counter this stigma, workers may not always be motivated to spontaneously challenge the stigma surrounding their occupation. Drawing on Stigma Theory (Yang et al. 2007), workers may make a choice about whether to challenge or not challenge occupational stigma as they respond to stigmatising discourses via interpersonal communication. Examining these spontaneous responses may offer valuable insights into workers’ authentic choices and attitudes, as these responses are given without external prompts or potential biases influencing their content. By analysing unprompted reactions, we can gain a clearer understanding of how workers genuinely perceive and manage occupational stigma in their daily interactions. This approach can reveal the nuanced ways in which ACWs challenge stigma, providing a more comprehensive picture of their discursive strategies.

### Challenging or not challenging occupational stigma in communication

Stigma theory postulates that individuals arrive at a mutual understanding and evaluation of their occupation socially through an exchange of meanings in language. Meisenbach and Hutchins (2020) suggest that individuals, upon receiving a stigmatising message, have the opportunity to (re)negotiate their interpersonal relationship with those who (re)produce stigma, via choosing to either challenge the discourse or not. This process can involve conformity and dissent, wherein people can agree or disagree with one another about what they are evaluating. Through this exchange, individuals may feel empowered to condemn or distance themselves from stigmatising messages.

Ashforth and colleagues (2007) argue that workers may be able to neutralise the impact/salience of “dirty work” by emphasising aspects of their work that are considered positive qualities. ACWs who discursively challenge stigma may feel empowered to change negative evaluations surrounding their occupation. They may discursively respond to stigmatising discourses by entertaining positive values about their occupation (e.g. “*working in aged care is devalued but it is fundamentally important to older adults and society”)*, which in turn may render negative values as less applicable (Mikolon et al. 2021, p.11). As a result, workers may help negate stigmatising discourses, through infusing positive value to negative evaluations targeted at this type of work and/or workers who perform this work. Alternatively, some people may not take the opportunity to challenge stigma, and instead choose to restate negative values about their occupation (e.g. “*working in aged care is devalued because it is considered a low status job”*; Manchha et al. 2021). In doing so, they reproduce common assumptions in the form of societal discourses that stigmatise aged care work and/or workers*.* ACWs’ choice to challenge or reproduce stigma are shaped by social and psychological factors. Some workers choose to conform and reproduce of stigmatising social discourses due to fear of negative repercussions and lack of supportive resources (Major et al. 2018).

### Relationship between ACWs’ discursive responses and mental health outcomes

ACWs may experience internalised stigma and/or psychological distress due to adverse social encounters, such as prejudicial attitudes and discrimination associated with the stigma surrounding aged care (Manchha et al. 2021). Some ACWs may address this stigma by challenging these negative perceptions, employing discourses that mitigate the psychological and social costs of working in a stigmatised occupation. Existing qualitative studies have investigated such discursive responses. McGuire and colleagues (2022) reported that nurses in residential care promote a skilled professional identity to challenge the outdated stereotypes of ACWs. Similarly, Clarke and Ravenswood (2019) found that health professionals shifted attention away from the work and instead highlighted the specialised skills required to care for older adults. Ostaszkiewicz et al. (2016) found that ACWs redefined incontinence care as "dignity work" to alter its negative connotations. Such discursive strategies might positively impact workers' mental health by enabling them to reframe the societal discourse surrounding their occupation in a more positive light. However, there is a notable gap in quantitative work that directly tests whether the use of such discursive strategies that challenge stigma is associated with better psychological wellbeing. The present study is the first to empirically test the relationship between discursive strategies and two mental health outcomes—internalised occupational stigma and psychological distress. Findings could offer valuable insights into whether internalised stigma and psychological distress differs as a function of whether ACWs spontaneously challenge the stigma associated with aged care, which could inform more effective stigma reduction strategies for this cohort. Based on the work above, we hypothesise:

#### Hypothesis 1

ACWs’ internalised occupational stigma will be lower for those who discursively challenge compared to those who do not challenge stigmatising discourses surrounding aged care.

#### Hypothesis 2

ACWs’ psychological distress will be lower for those who discursively challenge compared to those who do not challenge stigmatising discourses surrounding aged care.

## Methods

We employed a sequential mixed-methods approach adhering to Mixed-Methods Article Reporting Standards (MMARS). The study comprised three phases. In the first phase, we presented ACWs with a broad, open-ended question asking them to discuss how the stigma of aged care has affected their own attitudes towards working in aged care, thereby giving them the opportunity to either challenge or not challenge stigmatising discourses about their occupation. We conducted a language-based content analysis (APPRAISAL framework; Martin and White 2005) to systematically categorise workers’ discursive responses into two types – ‘challenge’ and ‘no challenge’. This allowed us to identify what proportion of ACWs proactively and spontaneously challenge stigmatising discourses associated with working in aged care via discursive responses. The second phase involved a quantitative analysis to test whether workers’ mental health outcomes (i.e. internalised occupational stigma, psychological distress) differ between those who employ different discursive responses (i.e. ‘challenge’ vs. ‘no challenge’). In the third phase, we further employed the APPRAISAL framework (Martin and White 2005) to identify the *types* of discourses that ACWs use to discursively challenge stigma surrounding their work (amongst those who chose to ‘challenge’).

### Participants and procedure

We used Prolific, an online participant recruitment platform to recruit people who worked in aged care. This system uses convenience sampling (e.g. any participant who meets the criteria is able to participate if they complete the survey before the quota is reached). Participants were eligible to participate in the study if they were over 18 years of age, lived in UK, USA, Australia or NZ and have worked in aged care in the past 4 weeks. We targeted these countries because they share similar systems of providing aged care (e.g. private and public models of care), allowing for more relevant and comparable analysis. The final sample consisted of 184 adults (78% females, aged 19–69 (*M*_age_ = 37.26, SD = 10.75 years, see Table 1 for demographic characteristics).

**Table 1:** Participant demographic characteristics

Demographic characteristics	N = 184	
	*n*	%
Gender		
Female^a^	145	78.80
Male	39	21.20
Country		
United Kingdom	124	67
United States	56	30
Australia	4	2
Highest educational level		
Some high school	5	2.70
High school	17	9.20
Diploma	15	8.20
Trade/technical/vocational training	33	17.90
Undergraduate degree	78	42.40
Postgraduate degree	36	19.60
Race and ethnicity		
White	135	73.40
Asian	25	13.50
Black/African American	17	9.20
Latinx	3	1.61
Multi-racial	3	1.61
Indigenous	1	0.50
Work role^b^		
Personal care	66	35.48
Allied health	56	30.40
Nursing	43	23.40
Management and administration	17	9.20
Auxiliary work	2	1.10
Work context^c^		
Hospital	82	–
Institutional care	48	–
Home care	48	–
Clinics	9	–
Community care	7	–
Respites	6	–
Retirement villages	2	–
Other (i.e. pharmacy, hospice)	2	–

^a^Representative of the gender disparity in this occupation

^b^Mean tenure was 7.40 years

^c^Workers may work at multiple contexts

Participants completed an online survey measuring internalised occupational stigma, psychological distress, and demographics. They were then provided with a definition of stigma (‘stigma is when other people think or feel that something has bad or undesirable qualities’) and included examples of aged care stigma (‘people have treated you unfairly based on negative stereotypes about aged care workers’, ‘people have treated you differently when it was revealed that you work in institutional aged care’) to prompt ACWs to reflect on their lived experiences. We tested this definition with participants in a pilot survey to confirm that participants understood what was being asked. Participants were then invited to provide a written response to an open-ended question: ‘*How does society's perceptions about stigma related to aged care impact your thoughts and feelings about working in aged care?*’ Participants received payment upon completing their survey via the Prolific platform. This study was granted ethics approval by The Human Research Ethics Committee of The University of Queensland.

## Analyses

### Phase 1: Content analysis using language-based criteria

The first author screened 186 responses to check whether participants provided a discursive response and excluded two participants from the data set. The remaining 184 responses varied from a few words to half a page. We then applied language-based criteria to differentiate ‘challenge’ responses from ‘no challenge’ responses. This criteria, informed by past occupational stigma studies (Meisenbach 2010), was employed to separate responses into tcategories according to whether the participant challenged societal stigmatising discourses related to aged care (‘challenge’) or did not (‘no challenge’). Another author independently coded responses with an inter-rater reliability indicating strong agreement (Kappa = 0.86). The team addressed any discrepancies and reached an agreement through consensus. We further drew on coding conventions from a linguistic framework (APPRAISAL, Martin and White 2005, Table 2) to understand the types of word choices that participants employed to challenge or not challenge stigma.

**Table 2:** Appraisal attitude subtypes used in this analysis

Attitude type	Attitude subtype	Examples from analysis
*Judgment* Expressions evaluating a person’s character based on ethics/moral sensibilities	*Normality:* Evaluating a person based on how distinguished they are compared to others (Positive *normality*: celebrated, Negative *normality*: unfortunate)	“I provide care to some of the most vulnerable people in this country…”
	*Capacity:* Evaluating a person based on their competence and capability	“People think that people who work within aged care are thick and haven’t got the skills to do anything else.”
	*Tenacity:* Evaluating a person based on their dependability and reliability	“I think many people think if you work in aged care you couldn’t find anywhere else to work. Or that you are lazy and don’t have the drive to apply to work anywhere else.”
	*Propriety:* Evaluating a person based on their ethics and morality	“There might be financial mistreatment by management in the aged care institutions and them taking advantage of hard and empathic workers.”
*Appreciation* Expressions ascribing social value to situations or processes	*Reaction:* Evaluating the value of a situation or process in terms of aesthetics (i.e. did I like it?)	“Many people have told me that working in aged care is dirty because you have to wipe poop and bathe elderly people.”
	*Composition:* Evaluating the value of a situation or process in terms of structure or form (i.e. how well do the parts fit together?)	“The staff numbers are so low and the time given to each individual requiring the care is so little shows how much stigma there is to elderly care.”
	*Valuation:* Evaluating the value of a situation or process in terms of what society deems as worthy and how people value them (i.e. is it worthwhile?)	“It makes me feel like people do not respect our job.”
*Affect* Expressions associated with emotional reactions	*Desire:* Feelings of longing (i.e. a person’s need or want)	“We all know that this is not a desirable role so it would be obvious that I had been unable to get a 'better' job and so had been forced to take this one.”
	*Happiness*: Feelings of cheer and affection (i.e. a person’s contentment)	“The work is depressing often and makes me feel sad.”
	*Security:* Feelings of confidence and trust (i.e. a person’s comfortability)	“I feel like family or friends of the clients watch and assess you unfairly and this can make you uneasy.”
	*Satisfaction*: Feelings of interest and pleasure (i.e. a person’s accomplishments)	“Other people believe my work is not very stimulating, it doesn't seem exciting.”

Through applying this framework to the textual responses, we found that participants who challenged stigmatising discourses used word choices that positioned the reader of their response positively towards aged care. Conversely, participants who did not attempt to challenge this stigma used word choices that positioned their reader negatively towards aged care. These language patterns enabled us to systematically classify statements that negate negative evaluations by attributing positive value about aged care as ‘challenge’ responses (e.g. *“aged care work is important to society”*), and statements that (re)produce commonly assumed negative evaluations about aged care as ‘no challenge’ responses (e.g. *“aged care work is devalued”*). We excluded any textual data that did not contain evaluative language and/or relevance to targets about aged care.

### Phase 2 Measures and quantitative analysis to determine differences in mental health outcomes

#### Types of discursive responses

Two types of discursive responses emerged from the language-based criteria. We created dummy variables to represent these responses (1 = *challenge*, 0 = *no challenge*)*.*

#### Internalised occupational stigma

A twelve-item measure comprised of items adapted from Ritsher et al. (2003) measured participants’ internalised occupational stigma (e.g. “I often feel disappointed in myself for working in aged care”; α = 0.83). Responses ranged from 1 = Strongly Disagree- 7 = Strongly Agree. A higher score indicated greater internalised occupational stigma.

#### Psychological distress

A six-item measure from the Kessler Psychological Distress scale (K6, Kessler et al. 2002) measured participants’ psychological distress (e.g. “Over the last 30 days, how often have you felt so depressed that nothing could cheer you up”; α = 0.91). Responses ranged from 1 = None of the time—5 = All of the time. A higher score indicated greater psychological distress. Means, standard deviations, correlations and the reliability of scales were calculated and independent-groups *t*-tests were conducted to determine whether internalised stigma and psychological distress differed significantly between workers who did or did not challenge the stigma of aged care in their discursive responses.

### Phase three: Discourse approach

We further grouped distinct combinations of recurring word choices in ‘challenge’ responses. This enabled us to identify specific discourses that ACWs employ to infuse positive value to their occupation. The first author coded the textual responses by applying standardised linguistic criteria as predefined in Martin and White (2005). Word choices were coded in terms of attitude types (see Table 1); (1) *judgment* (evaluations about an entity’s character attributes)- *normality* (i.e. how distinct a person is to others), *capacity* (i.e. how competent and skilled a person is), *tenacity* (i.e. whether someone is dependable and committed), *veracity* (i.e. whether someone is trustworthy and honest), and *propriety* (i.e. a person’s ethics or moral sensibilities); (2) *appreciation* (ascribing social value to entities/systems)-*reaction* (i.e. impressions of an object/process’s quality/impact), *composition* (i.e. perception of order/structure) and *valuation* (i.e. ascribed social worth); (3) *affect* (construed emotional responses) in terms of *desirability* (i.e. feelings of longing)*, happiness* (i.e. contentment)*, security* (i.e. comfort) and *satisfaction* (i.e. pleasure). Evaluations were then classified according to polarity (*negative* vs *positive)* and targets (*entities/systems in aged care)-* (a) care recipients (i.e. beneficiary of aged care services), (b) ACWs (i.e. paid employees of the aged care sector), (c) aged care providers (i.e. managers of aged care institutions), (d) aged care work activities (e.g. health/personal care/social assistance), (e) working conditions (e.g. pay/workload), (f) institutions (i.e. aged care facilities). Two different groups of researchers independently checked the coding to ensure the meaning of the sentences were interpreted correctly. Then, we identified combinations of attitudinal patterns (i.e. combinations of attitude types, polarity, targets).

## Results

### Phase 1 ‘Challenge’ vs ‘no challenge’ discursive responses

We differentiated ‘challenge’ discursive responses from ‘no challenge’ responses using language-based criteria to examine whether ACWs’ proactively and spontaneously challenge stigmatising discourses associated with working in aged care via discursive responses.

#### ‘Challenge’: infusing positive values into negative evaluations about their occupation

Participants who employed ‘challenge’ discursive responses (*n* = 95; 52%) entertained positive evaluations about their work by shutting down negative evaluations (akin to *reframing* from Ashforth and Kreiner 1999). Some participants countered occupational stigma by firstly acknowledging established negative evaluations about aged care and then explicitly demonstrating a shift in position using conjunctions (i.e. *‘but’, ‘however’*) to reject unfavourable opinions about aged care*; “some of my co-workers do leave a lot to be desired but there are really caring individuals that care for the elderly.”* (Female, 37, Allied health professional).

We also found that ACWs chose to open up conversations about non-stigmatising aspects of aged care (akin to *recalibrating* or *refocusing* from Ashforth and Kreiner 1999). For example,

“*My friends often ask me why do I do it? Why do I look after elderly people when all it is cleaning up human waste and adult babysitting. This often makes me angry as I think working with elderly people is great, yes sometimes a little frustrating and it seems like the routine is always the same but its honestly great! There is more to working in aged care than incontinence and dementia, it is looking after people at some of their most vulnerable times.”* (Female, 24, Nurse).

#### ‘No challenge’: (re)producing negative evaluations about their occupation

A relatively large number of workers (*n* = 89; 48%) chose not to challenge stigmatising discourses, and instead employed discursive responses that (re)produced negative evaluations about aged care in three ways. First, participants construed unfavourable character judgments about entities in aged care, for example, “*society thinks that individuals in aged care are incompetent and don’t care about the residents most of the time”* (Female, 30, Allied health professional). Second, participants recalled the low social value (i.e. status) and/or negative emotional reactions associated with their work: “*our job is low paid and the work we do is very emotionally draining*” (Female, 40, Allied health professional). Third, some participants did not attempt to shift negative values that construed this occupation as stigmatised in the first place; for instance, “*I am often asked if I am mad to work in a poorly paid job to wipe bottoms and feed people, but this has no impact on my thoughts and feelings as they did not ask to get old or need assistance…*” (Female, 50, Personal care worker). This response is similar to *refocusing* from Ashforth and Kreiner (1999) in redirecting attention away from occupational stigma. However, as these participants did not attribute positive value to their occupation, these responses are deemed conceptually different to ‘challenge’ responses. In line with our criteria, these responses tacitly (re)produce negative values about their occupation and thus, are conceptualised as a ‘no challenge’ response.

### Phase 2 Differences in mental health outcomes as a function of discursive responses

The independent-groups *t*-tests revealed that workers who employed ‘challenge’ discursive responses reported lower internalised occupational stigma (*M* = 3.21, SD = 0.83) compared to those who employed ‘no challenge’ responses, (*M* = 3.70, *SD* = 0*.*99), *t*(182) = 3.62, *p* < 0.001, *d* = 0.54, 95% CI [0.24,0.83], supporting hypothesis 1. Workers who challenged stigmatising discourses also reported significantly lower psychological distress (*M* = 1.86, SD = 0.78) compared to those who did not challenge (*M* = 2.16, SD = 0.99), t(182) = 2.29, *p* = 0.023, *d* = 0.34, 95% CI [0.04,0.63], supporting hypothesis 2. Table 3 presents the means, standard deviations, and correlations between the variables of interest.

**Table 3:** Means, standard deviations, and zero-order correlations among the hypothesised variables and outcomes

	M	SD	1	2	3	4	5	6
1. Age	37.29	10.80	–					
2. Gender (female)^a^	–	–	.12	–				
3. Tenure (yrs)	7.38	7.34	.58^**^	.12	–			
4. Type of discursive response (challenge)^b^	–	–	.09	.09	.10	–		
5. Internalised occupational stigma	3.44	0.94	.11	.09	.04	.26^**^	–	
6. Psychological distress	2.00	0.90	.24^**^	.05	.20^**^	.17^*^	.37^**^	–

M: mean SD: Standard deviation

^*^p < .05. **p < .01

^a^ Gender was coded 0 = Female, 1 = Male, point-biserial correlation

^b^Type of discursive response was coded 1 = ‘challenge’, 0 = ‘no challenge’, point-biserial correlation

### Phase 3: Discourses

We identified four distinct discourses that were employed by ACWs to infuse positive value into negative evaluations about ACWs and work; (1) *Hard Workers*, (2) *Esteemed Workers*, (3) *Essential Work,* and (4) *Rewarding Work*.

#### Discourse 1- Hard workers (n = 25)

In this discourse, ACWs focus on challenging negative discourses by emphasising their dedication and exceptional qualities. Participants in this group aim to shift perceptions by spotlighting the hard work and personal commitment (*positive tenacity*) inherent in their roles. For example, “*in my time working in care I have met some amazing people who would go above and beyond to help anyone.*” (Female, 28, Personal care worker). This discourse highlights how workers use their own dedication and positive experiences to challenge generalised negative perceptions of their profession, “*they are often treated with disdain by residents and their families yet continue to show commitment and empathy in their work* (Female, 44, Allied health professional).

#### Discourse 2- Esteemed workers (n = 4)

This discourse is characterised by a dual focus on recognising the essential contributions of ACWs while acknowledging the low status often associated with their roles. Participants using this discourse emphasise their significant societal role (*positive valuation*), despite the general underappreciation they face. For instance, one participant noted, “*it can be frustrating to be undervalued but at the end of the day the patient (usually) appreciates what you are doing*” (Male, 32, Allied health professional). This discourse demonstrates how ACWs seek to affirm their valuable contributions while simultaneously distancing themselves from the negative status typically attributed to their profession, “*I think that patients and colleagues value [ACW] very highly, but 'outsiders' make assumptions and judgements* (Female, 53, Nurse).

#### Discourse 3- Essential work (n = 17)

The "Essential Work" discourse involves a more comprehensive approach to challenging stigma by addressing multiple facets of the stigmatised occupation (e.g. role of workers, work activities, working conditions). Participants in this group highlight the crucial nature and the benefits of aged care work (*positive valuation*) to both clients and society, despite existing negative views, “*I know what an important and valuable job I do, and it comes with many benefits to both myself, my colleagues, and the service users we care for*” (Female, 39, Allied Health Professional). This discourse underscores how ACWs advocate for a unified view of their work's significance and positive impact, challenging the negative stereotypes associated with their profession, “*people tend to think aged care work is dirty or lowly, a last resort. Even though it's essential and really makes a difference to people”* (Female, 33, Allied Health Professional).

#### Discourse 4- Rewarding work (n = 11)

The "Rewarding Work" discourse focuses on normalising the positive emotional experiences and job satisfaction associated with aged care work. Participants using this discourse aim to emphasise the intrinsic satisfaction and personal fulfilment derived from their work, despite the prevalent stigma. For example, one participant shared, “*I enjoy working in aged care as I find it a rewarding experience. In particular supporting people at the end of their lives and their relatives…*” (Male, 40, Nurse). This discourse highlights how ACWs frame their work as deeply fulfilling and rewarding, thereby challenging the negative stereotypes and emphasising the positive emotional aspects of their profession, “*people think individuals who work at elderly care don't not have the option to go anywhere but, in reality, its satisfying job* (Female, 27, Personal care worker). Table 4 presents the combinations of targets and attitude types underpinning each discourse.

**Table 4:** Discourses that infuse positive value into negative evaluations about working in aged care

Target	Negative values and attitude types	Example	Target	Positive values and attitude types	Example	Frequency
*Discourse 1: Hard Workers*						*n* = 25
Workers	Negative *Capacity*	People think aged care work sometimes is for under educated [-capacity] people…	Workers	Positive *Tenacity*	… ACWs work hard [+ tenacity] to help provide great care. (ACW_103, Female, 32 Nurse)	*n* = 4
	Negative *Valuation*	They are often treated with disdain [-valuation] by residents and their families…	Workers	Positive *Tenacity*	…yet continue to show commitment and empathy [+ tenacity] in their work. (ACW_94, Female, 44 Allied health professional)	*n* = 8
Work activities	Negative *Reaction*	Some people think its just wiping butts [-reaction]…	Workers	Positive *Tenacity*	…but that’s not all. All my colleagues trained to work with elders and wouldn’t ever leave their job [+ tenacity]. (ACW_109, Female, 32, Allied health professional)	*n* = 4
	Negative *Valuation*	There is a belief it’s a job anyone can do [-valuation]…	Workers	Positive *Tenacity*	…but not a job everyone sticks at. It requires such mental strength [+ tenacity] to do. (ACW_58, Female, 25, Allied health professional)	*n* = 3
Working conditions	Negative *Composition*	Nursing assistants may feel that there is a lot of physical workload [-composition]…	Workers	Positive *Tenacity*	when it actually involves resilience and strength [+ tenacity] due to dealing with emotional aspects as well. (ACW_170, Female, 24, Personal care worker)	*n* = 3
	Negative *Valuation*	Most of the jobs are low paying [-valuation] It makes me a bit embarrassed at times, because most people have a pretty good idea that I don't make much considering what I do [-valuation]...	Workers	Positive *Tenacity*	… I think some people are more understanding and think it's great if you are actually a caring [+ tenacity] and honest worker, and then it makes me feel good about my job. (ACW_11, Female, 37, Personal care worker)	*n* = 3
*Discourse 2: Esteemed Workers*						*n* = 4
Workers	Negative *Valuation*	…nobody really mentions [-valuation] the health care/nursing assistants who…	Workers	Positive *Valuation*	… actually do a lot of really important [+ valuation] things for patients. (ACW_121, Female, 24, Personal care worker)	*n* = 4
*Discourse 3: Essential Work*						*n* = 17
Workers	Negative *Valuation*	I think that people believe that working in aged care can be categorised as being a maid [-valuation] …	Work activities	Positive *Valuation*	…the people who work there however believe that it is the most humane and empathic [+ valuation] job one can do. (ACW_52, Female, 28, Personal care worker)	*n* = 3
Work activities	Negative *Reaction*	I think society views working in aged care as a 'dirty job' cleaning up old people, though sometimes it isn't pleasant [-reaction]…	Work activities	Positive *Valuation*	…it is a needed area of society. [+ valuation] (ACW_72, Male, 35, Nurse)	*n* = 6
	Negative *Valuation*	I feel embarrassed because most people don't think much of aged care work [-valuation]	Work activities	Positive *Valuation*	…even though it is a necessary [+ valuation] task. (ACW_31, Female, 31, Allied health professional)	*n* = 2
Working conditions	Negative *Composition*	Workers are often expected to service a totally unreasonable [-comp] number of patients during their shifts…	Work activities	Positive *Valuation*	… yet do absolutely vital [+ valuation] jobs. (ACW_19, Female, 53, Allied health professional)	*n* = 2
	Negative *Valuation*	I think a big part of the stigma surrounding working in aged care comes from the low pay [-valuation] and therefore status [-valuation] people receive for…	Work activities	Positive *Valuation*	… what is actually a very rewarding and important [+ valuation] job. (ACW_139, Female, 37, Personal care worker)	*n* = 4
*Discourse 4: Rewarding Work*						*n* = 11
Care recipients	Negative *Normality*	I take pride in helping those who are vulnerable and frail. [-normality]	Work activities	Positive *Satisfaction*	…I enjoy [+ satisfaction] the time spent with the people who I work with even if society views them differently. (ACW_40, Male, 28, Allied health professional)	*n* = 3
Workers	Negative *Valuation*	People think individuals who work in elderly care don't have the option to go anywhere [-valuation]	Work activities	Positive *Satisfaction*	…but, in reality, its satisfying job [+ satisfaction]. (ACW_27, Female, 27, Personal care worker)	*n* = 2
Work activities	Negative *Satisfaction*	Other people believe my work is not very stimulating, it doesn't seem exciting [-satisfaction]	Work activities	Positive *Satisfaction*	…however I find it very rewarding. [+ satisfaction] (ACW_44, Female, 41, Allied health professional)	*n* = 2
Working conditions	Negative *Composition*	The tasks are difficult [-composition]	Work activities	Positive *Satisfaction*	… but rewarding [+ satisfaction]. (ACW_199, Female, 26, Management and admin)	*n* = 2
	Negative *Valuation*	Because the pay is low [-valuation]	Work activities	Positive *Satisfaction*	…it tends to mean that the people who work in the industry do it because they love what they do [+ satisfaction] and care for people in need. This has definitely been my experience of colleagues I have worked with. (ACW_139, Female, 37, Personal care worker)	*n* = 2

## Discussion

This mixed-methods study examined how ACWs discursively challenged or did not challenge stigmatising discourses surrounding their occupation and explored the association between these responses and mental health indicators. We found that when provided with an open question, a large proportion of ACWs did not proactively challenge stigmatising discourses. For those workers who did challenge stigmatising discourses, discursive responses exhibited linguistic patterns consistent with theoretical descriptions in the occupational stigma literature (Ashforth et al. 2007). For instance, participants shut down negative evaluations by emphasising positive value (e.g. ‘priceless work’). Additionally, participants emphasised non-tainted aspects of work that are important to them (e.g. helping people, career opportunities; Bosmans et al. 2016). While our findings support Ashforth and Kreiner’s (1999) theorising about occupational ideologies, our analytical method allows us to systematically identify detailed textual evidence based on established linguistic criteria. For example, our study offers insight into the frequency at which ACWs challenge or do not challenge occupational stigma, and successful discourses that workers employ in communication that may be able to reduce the psychological burden of occupational stigma. Findings suggest that workers who were motivated to challenge stigma displayed lower internalised occupational stigma and psychological distress.

In line with Mikolon et al. (2021), we found that workers attempted to transform the meaning of their occupation through infusing positive value in multiple ways. ACWs strived to negate stigma attributed to two targets in the aged care context through four distinct discourses that invoked a range of positive attitude types: ACWs (i.e. *tenacity*, *valuation*) and aged care work (i.e. *valuation* and *satisfaction*). This finding extends existing knowledge about stigma management, which has previously assumed that people infuse positive value to their work in a single way through promoting social value. We provide evidence that suggests workers also infuse positive value about people’s character attributes and emotional responses. These nuances in infusing a range of positive value based on attitude types have not been examined in past studies and may benefit from additional investigation.

### Implications for theory

We extend existing theory by recognising that people can challenge stigmatising discourses through infusing positive value, or they can choose not to challenge occupational stigma, instead (re)producing negative evaluations. This takes an alternative social approach to past studies that assumed workers cope with occupational stigma introspectively (e.g. Soral et al. 2021). Furthermore, research has predominantly framed workers who employ ‘no challenge’ discursive responses negatively given that it is a passive response to stigmatising discourses that does not attempt to redress them (Bosmans et al. 2016). We found support for this, such that internalised occupational stigma and psychological distress was lower in workers who challenge than those who do not challenge stigmatising discourses. This suggests that challenging occupational stigma, by infusing positive value into the work, may be important to protect workers’ psychological wellbeing from the threat associated with being in a stigmatised occupation.

However, it is also evident that not all workers have equal access to those discursive strategies that can enable them to challenge the stigma. It is possible that social positioning may play a role in determining whether workers can effectively challenge stigma. Workers with higher social capital (e.g. strong support networks) may be better equipped to reframe stigmatising narratives. These individuals often have greater access to resources and opportunities where they can assert positive aspects of their work, thereby mitigating the impact of stigma. Conversely, workers with limited social capital may struggle to find or create such opportunities, leaving them more vulnerable to the negative effects of stigma. This disparity underscores the need for targeted interventions that address these gaps.

### Implications for practice and policy

By taking a social approach, our study offers insight for developing guidelines for: (1) language-based interventions that champion successful discursive responses for people working in stigmatised occupations, (2) training for navigating responses to stigmatising discourses, and (3) reconceptualising public messaging at a system-level. We argue that practical interventions seeking to address occupational stigma require discursive interventions. This study proposes a new trajectory of language-based interventions that do not merely seek to change workers’ mindset about their occupation in isolation amidst a hostile environment. Instead, it functions to provide workers with successful discursive resources to help them engage meaningfully with those they are interacting with who may hold stigmatising opinions about aged care (e.g. society, relatives of care recipients, friends/family of workers, formal colleagues). We propose not just training workers to repeat prescribed sentences, but rather guiding them to craft responses from their own personalised experiences to support positive evaluations about their occupation. Training could be offered during initial education and in continuing professional development to prepare people to challenge stigmatising discourses to improve their psychological wellbeing and potentially, organisational outcomes (e.g. retention, productivity; Zhang et al. 2021).

Furthermore, system-level interventions (i.e. media campaigns) may integrate findings from our study to design more targeted and evidence-based public messaging. Targeted public messaging can play a crucial role in transforming and deconstructing stigmatising stereotypes about ACWs by emphasising their positive contributions and correcting misconceptions. Strategies include showcasing personal stories that highlight the compassion and professionalism of ACWs and broadening the representation of specific pairings of positive and negative value (see Table 4) to frame aged care as a vital societal resource. Regular monitoring of the social perceptions about aged care (e.g. reviewing media coverage) could track attitudinal changes in the target audience.

### Limitations and agenda for future research

We acknowledge a limitation is obtaining discursive responses through a short survey response, which may not provide a comprehensive understanding about how discursive responses are used dynamically in conversation. Although, this study identified recurring language patterns that may be indicative of the habitual responses that individual participants generally use, we recognise that this study did not examine whether a combination of discursive responses may occur throughout a longer stretch of dialogue. This can be clarified through further research into discursive responses constructed in contextualised conversations. Due to constraints related to the number of responses and limited statistical power, we were unable to test which types of challenging discourses best predict lower levels of internalised stigma and psychological distress. Future studies could investigate the impact of different types of challenging discourses using experimental designs or larger sample sizes. We also suggest that future studies explore the different and complex ways in which occupational stigma surrounding aged care is socially constructed across aged care contexts (i.e. institutional settings vs at-home services).

## Conclusions

This study sheds light on the nuanced ways in which ACWs navigate and respond to occupational stigma through discourse. Our findings reveal a significant difference in ACWs engagement with stigmatising narratives, with many choosing not to challenge these discourses directly. However, for those who do challenge stigma, the use of positive-value-infused language was a robust strategy. Engaging in discursive challenges also correlates with lower levels of internalised stigma and psychological distress among ACWs. This highlights the importance of providing workers with the tools to effectively counteract negative stereotypes and reinforces the need for language-based interventions to better support workers in navigating and mitigating the impact of occupational stigma.

## Supplementary Information

Below is the link to the electronic supplementary material.

Supplementary material

## Data Availability

Requests for access to data and materials can be made with the corresponding author who can facilitate a data use agreement as appropriate.
