# Supplementary material for: Challenging aged care stigma through communication: discursive responses to stigmatising discourses about aged care work and implications for workers’ mental health
Source: Eur J Ageing. 2025 Apr 3;22(1):14. doi: 10.1007/s10433-025-00844-2 (PMC11968573; doi:10.1007/s10433-025-00844-2)
Supplement: Supplementary file 1 — Supplementary material [file 10433_2025_844_MOESM1_ESM.docx]

**Supplementary material**

**Descriptive statistics for discourse strategies and outcomes**

| Discourse strategies | Internalised occupational stigma | | Psychological distress | |
| --- | --- | --- | --- | --- |
|  | **M** | **SD** | **M** | **SD** |
| *Discourse 1: Hard Workers* | 3.29 | 1.00 | 2.01 | 0.73 |
| *Discourse 2: Esteemed Workers* | 3.73 | 1.53 | 2.08 | 0.44 |
| *Discourse 3: Essential Work* | 3.39 | 0.95 | 1.89 | 0.98 |
| Discourse 4: *Rewarding Work* | 3.35 | 0.86 | 1.86 | 0.98 |
| Uncategorised | 3.14 | 0.75 | 1.79 | 0.76 |

Note: M: mean SD: Standard deviation
